# Adiponectin modulates oxidative stress-induced mitophagy and protects C2C12 myoblasts against apoptosis

**DOI:** 10.1038/s41598-017-03319-2

**Published:** 2017-06-09

**Authors:** Yinghui Ren, Yan Li, Jun Yan, Mingkun Ma, Dongmei Zhou, Zhenyi Xue, Zimu Zhang, Hongkun Liu, Huipeng Yang, Long Jia, Lijuan Zhang, Qi Zhang, Shuqin Mu, Rongxin Zhang, Yurong Da

**Affiliations:** 1 Department of Immunology, Research Center of Basic Medical Sciences, Key Laboratory of Immune Microenvironment and Diseases of Educational Ministry of China, Tianjin Medical University, Tianjin, China; 2 Tianjin Institute of Animal husbandry and veterinary, Tianjin Academy of Agricultural Science, Tianjin, 300381 China; 3 College of Chemical Engineering, Tianjin University, Tianjin, 300072 China; 4 Second Affiliated Hospital of Tianjin University of Traditional Chinese Medicine, Tianjin, China; 5 Laboratory of Immunology and Inflammation, Guangdong Pharmaceutical University, 510000 Guangzhou, China

## Abstract

Adiponectin (APN), also known as apM1, Acrp30, GBP28 and adipoQ, is a circulating hormone that is predominantly produced by adipose tissue. Many pharmacological studies have demonstrated that this protein possesses potent anti-diabetic, anti-atherogenic and anti-inflammatory properties. Although several studies have demonstrated the antioxidative activity of this protein, the regulatory mechanisms have not yet been defined in skeletal muscles. The aim of the present study was to examine the cytoprotective effects of APN against damage induced by oxidative stress in mouse-derived C2C12 myoblasts. APN attenuated H_2_O_2_-induced growth inhibition and exhibited scavenging activity against intracellular reactive oxygen species that were induced by H_2_O_2_. Furthermore, treating C2C12 cells with APN significantly induced heme oxygenase-1 (HO-1) and nuclear factor-erythroid 2 related factor 2 (Nrf2). APN also suppressed H_2_O_2_-induced mitophagy and partially inhibited the colocalization of mitochondria with autophagosomes/lysosomes, correlating with the expression of Pink1 and Parkin and mtDNA. Moreover, APN protected C2C12 myoblasts against oxidative stress-induced apoptosis. Furthermore, APN significantly reduced the mRNA and protein expression levels of Bax. These data suggest that APN has a moderate regulatory role in oxidative stress-induced mitophagy and suppresses apoptosis. These findings demonstrate the antioxidant potential of APN in oxidative stress-associated skeletal muscle diseases.

## Introduction

Oxidative stress, which is the pathological basis of many chronic diseases, results from disturbance of the balance between free radicals and antioxidant defenses^1^. Free radicals are generated in the form of reactive oxygen species (ROS), including short-lived superoxide anions, more stable hydrogen peroxide molecules and highly reactive hydroxyl radicals^2, 3^. ROS can be generated during mitochondrial oxidative phosphorylation or by several enzymes, including NADPH oxidases, xanthine oxidases and lipoxygenases. At low levels, ROS act as second messengers of signal transduction and participate in cellular signaling processes. Conversely, excessive ROS can lead to destructive and irreversible damage to all cellular constituents, such as nucleic acids, proteins and lipids^4^; therefore, ROS levels must be tightly regulated^5^.

Mitochondria have major roles in many cellular processes, including ATP production, fatty acid oxidation, cell survival, apoptosis, and necrosis^6, 7^. The accumulation of ROS within mitochondria can cause mitochondrial DNA mutations, lipid peroxidation and the opening of mitochondrial membrane channels including inner membrane anion channels (IMACs) and mitochondrial permeability transition pores (MPTPs). The opening of these channels leads to a transient increase in ROS generation referred to as ROS-induced ROS release (RIRR) and a decrease in mitochondrial membrane potential^4^. Furthermore, the opening of the MPTPs increases the permeability of mitochondria, which might cause a decrease in ATP concentrations and mitochondrial swelling^8–10^. The elimination of damaged mitochondria is essential for ensuring efficient energy supply and maintaining mitochondrial quality. There are two major catabolic processes by which dysfunctional mitochondria are degraded: one is the ubiquitin-proteasome system for eliminating mitochondrial outer membrane proteins, and the other is the autophagy-lysosome pathway for degrading mitochondria as whole organelles^11, 12^. The latter process, also known as mitophagy, selectively excludes damaged mitochondria via a specific autophagic pathway^13^.

Autophagy involves catabolism of cellular constituents, including organelles, the cytosol and proteins; this process occurs through the encapsulation of cellular components into a double-membrane structure termed an autophagosome^14, 15^. Two types of macroautophagy have been identified. In nutrient-deficient circumstances, non-selective autophagy supplies cells with essential metabolites and energy until nutrients can be obtained from the environment^16^. By contrast, under nutrient-rich conditions, selective autophagy mediates the removal of damaged or excess organelles, such as peroxisomes^17^, endoplasmic reticulum (ER)^18–21^ and mitochondria^22^, and accumulating evidence suggests that preferential autophagic processes are induced in response to ROS^4^. Mitophagy has been proposed to decrease potential oxidative damage due to dysfunctional mitochondria. However, recent reports have shown that a form of autophagic cell death is activated in response to oxidative stress^23^.

Adiponectin (APN), also known as 30-kDa adipocyte complement-related protein, is a hormone that is abundantly secreted by adipocytes^24^. Several experimental studies have suggested that APN exhibits insulin-sensitizing^25^, anti-atherogenic^26^ and anti-inflammatory properties^27, 28^ and can exert a modulatory effect on oxidative stress^29, 30^. In addition, it has been shown that APN attenuates oxidative stress-induced autophagy in cardiomyocytes^30^. While imbalance between ROS production and elimination results in oxidative stress, which has been implicated in numerous skeletal muscle diseases, including age-related loss of muscle quantity(sarcopenia^31, 32^), age-related loss of muscle strength (dynapenia^33^), early-onset myopathies^34^ and many muscular dystrophies^35, 36^, the mechanisms underlying the impairment have not been elucidated. In the present study, a specific ROS, H_2_O_2_, was both sufficient and essential for inducing oxidative stress^4^. Although pathophysiological levels of H_2_O_2_ increase oxidative stress and apoptosis in mouse-derived C2C12 myoblasts^3^, little is known regarding the effects of APN on the pathophysiological processes of ROS-induced autophagy. Therefore, we sought to validate the hypothesis that APN modulates the pathophysiological levels of ROS-induced autophagy in C2C12 myoblasts and to elucidate the underlying mechanism. Our results indicate that APN protects skeletal muscles from oxidative stress-induced damage.

## Results

### APN reduces H_2_O_2_-induced C2C12 cytotoxicity

**Figure 1:**
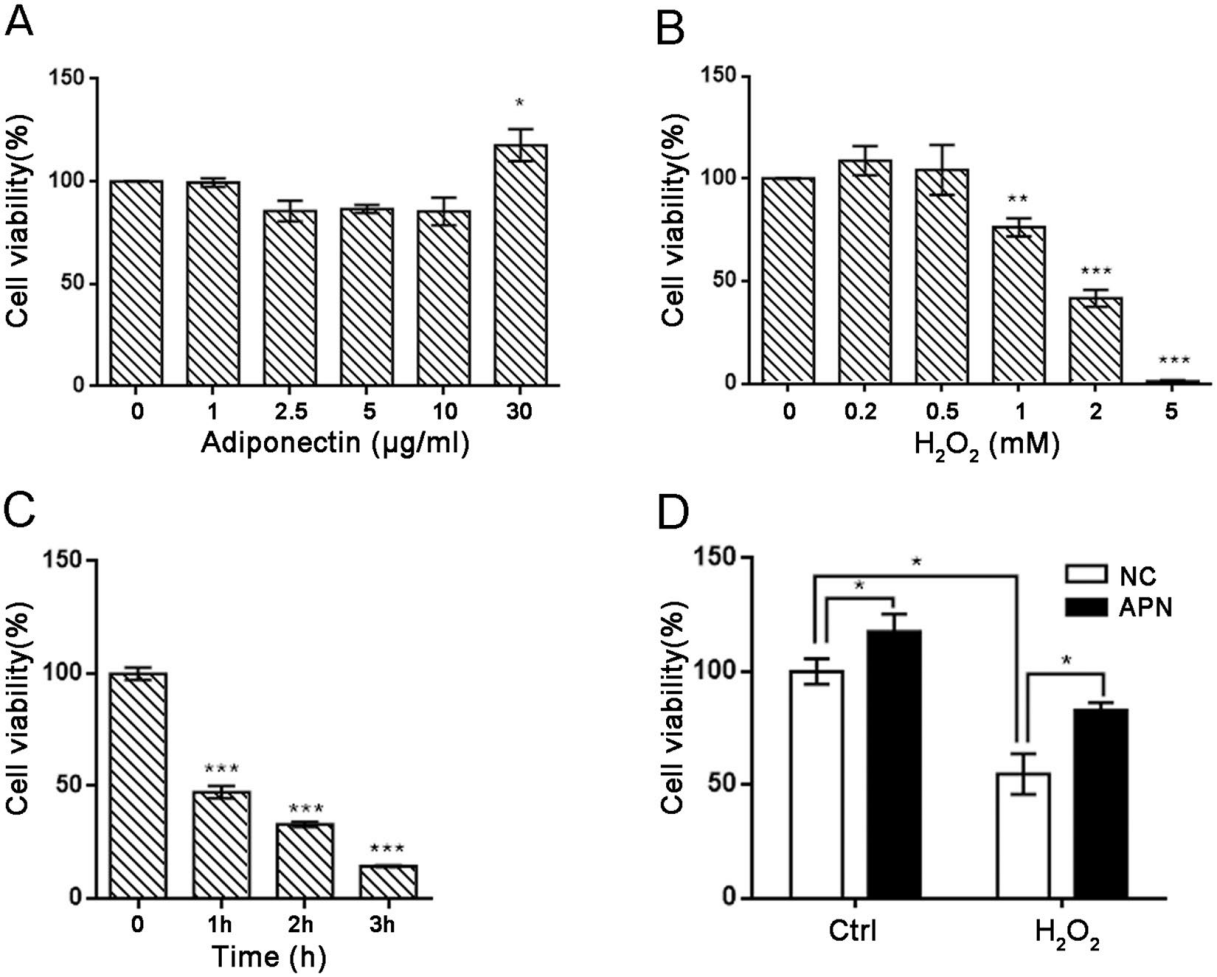
APN attenuates H_2_O_2_-induced growth inhibition in C2C12 cells. Cells were treated with various concentrations of APN for 24 h (**A**) or pretreated with 30 μg/mL APN for 24 h and then incubated with or without 5 mM H_2_O_2_ for 1 h (**D**). Dose-dependent (**B**) and time-dependent (**C**) inhibition of cell growth by H_2_O_2_ was evaluated using a Cell Counting Kit-8. The data are expressed as percentages of the mean values ± SEM of experiments performed in triplicate (*p < 0.05, **p < 0.01, ***p < 0.001).

Cells were first treated with a wide range of APN concentrations (1 to 30 μg/mL) for 24 h to determine the effect of APN on the viability of C2C12 cells. APN treatment at concentrations up to 30 μg/mL did not result in any cytotoxic effects, whereas cell viability increased at the concentration of 30 μg/mL (Fig. 1A). Cell viability dose-dependently decreased at concentrations from 1 to 5 mM H_2_O_2_ (Fig. 1B) and time-dependently decreased from 1 to 3 h (Fig. 1C). Therefore, 30 μg/mL APN was chosen as the optimal dose for studying the cytoprotective effect of APN against H_2_O_2_-induced cell damage. To examine the protective effect of APN on H_2_O_2_-induced cytotoxicity, C2C12 cells were treated with 30 μg/mL APN 24 h prior to H_2_O_2_ treatment, and then cell viability was measured. Our results indicated that treatment with 5 mM H_2_O_2_ alone reduced cell viability by approximately 50% after 1 h. Cytotoxicity was induced by 5 mM H_2_O_2_ for 1 h; other study reports 1 mM H_2_O_2_ for 6 h^3^. However, pretreatment with APN significantly protected cells against the H_2_O_2_-induced reduction in cell viability (Fig. 1D), indicating that the exposure of C2C12 cells to APN conferred a protective effect against oxidative stress.

### APN attenuates H_2_O_2_-induced ROS generation and apoptosis in C2C12 cells

**Figure 2:**
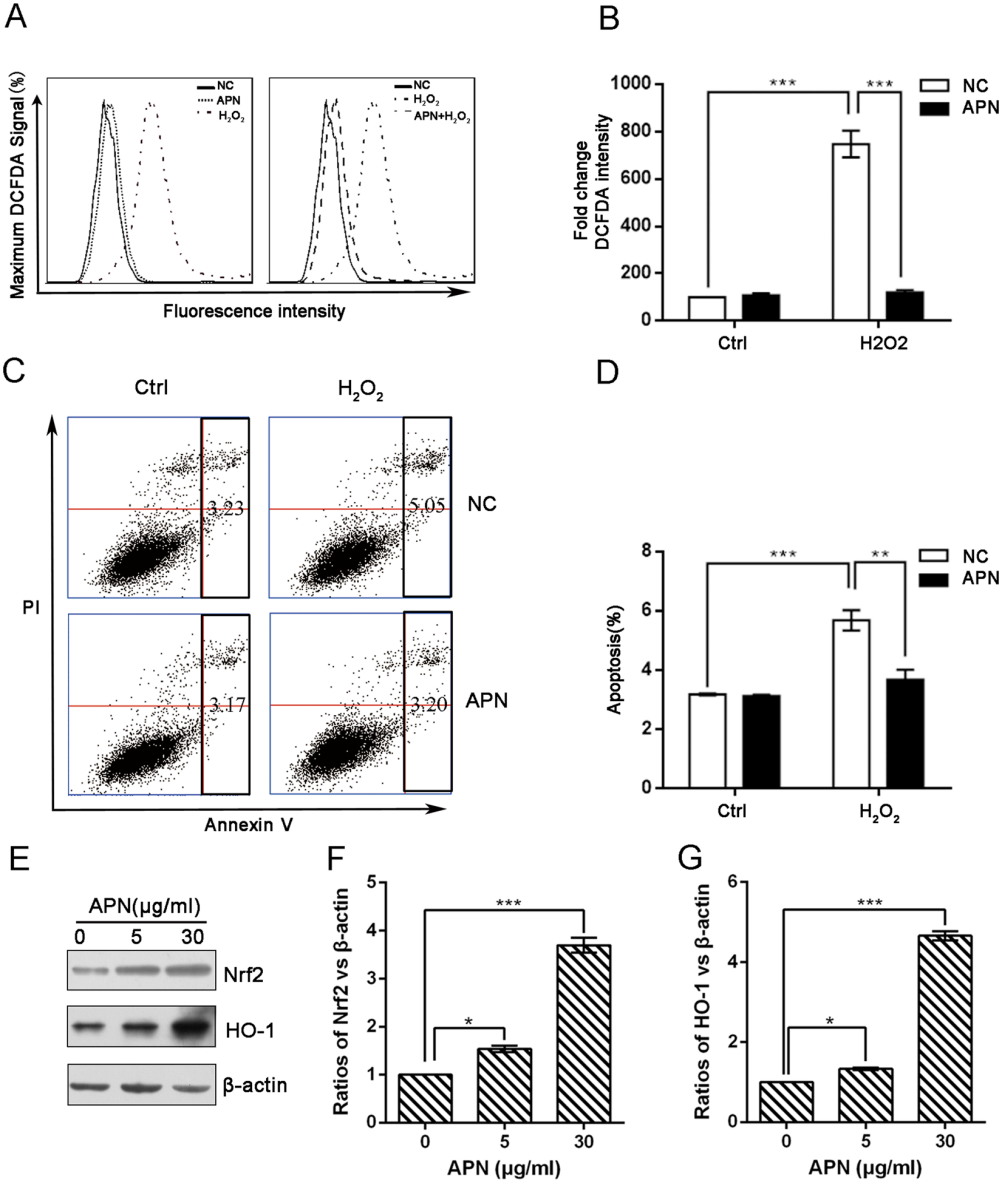
APN inhibits H_2_O_2_-induced ROS generation and apoptosis in C2C12 cells. C2C12 cells were pretreated with 30 μg/mL APN for 24 h and then incubated with or without 5 mM H_2_O_2_ for 0.5 h. (**A**) To monitor ROS production, cells were incubated at 37 °C in the dark for 30 min with fresh culture medium containing 10 μM DCFH-DA. ROS generation was measured using a flow cytometer. (**C**) Cells were also stained with annexin V-FITC and propidium iodide (PI), and the percentages of apoptotic cells were then analyzed via flow cytometric analysis. (**B**,**D**) The results are presented as the mean ± SEM values obtained from three independent experiments (**p < 0.01, ***p < 0.001). (**E**) Induction of Nrf2 and HO-1 expression by APN in C2C12 cells. The C2C12 cells were incubated with various concentrations of APN for 24 h. Image J densitometric analysis of the Nrf2/β-actin ratios (**F**) and HO-1/β-actin ratios (**G**) from the immunoblots is shown (data from three independent experiments) (*p < 0.05, ***p < 0.001).

We next investigated whether APN affected intracellular ROS generation due to H_2_O_2_ treatment using a 2′,7′-dichlorodihydrofluorescein diacetate (DCFH-DA) assay. As previously reported, ROS levels increased in H_2_O_2_-treated cells compared with untreated cells^37^. However, ROS levels were significantly inhibited in the presence of APN (Fig. 2A,B). To further evaluate the cytoprotective effects of APN that resulted from the prevention of H_2_O_2_-induced apoptosis, the frequency of apoptotic cells was detected by flow cytometry. The results showed that the treatment of C2C12 cells with APN prior to H_2_O_2_ exposure strongly protected the cells against apoptosis (Fig. 2C,D). These results indicate that oxidative stress-induced apoptosis is mediated by ROS generation and that APN exerts a potent ROS scavenging effect, preventing H_2_O_2_-induced apoptosis. As nuclear factor-erythroid 2 related factor 2 (Nrf2) is a master antioxidant transcription regulator^38, 39^, we evaluated the regulatory antioxidant potential of APN on Nrf2 protein expression by Western blotting. As shown in Fig. 2E,F, C2C12 cells exposed to various concentrations of the APN caused a concentration-dependent increase in Nrf2 expression compared with that in the control group. Furthermore, expression of hemeoxygenase-1 (HO-1), which is downstream molecules of Nrf2, also increased following APN treatment in a concentration-dependent manner (Fig. 2E,G). These data indicate that activating the Nrf2/HO-1 pathway is closely associated with the APN cytoprotective mechanism in C2C12 cells.

### APN restores the mitochondrial membrane potential inhibited by H_2_O_2_

**Figure 3:**
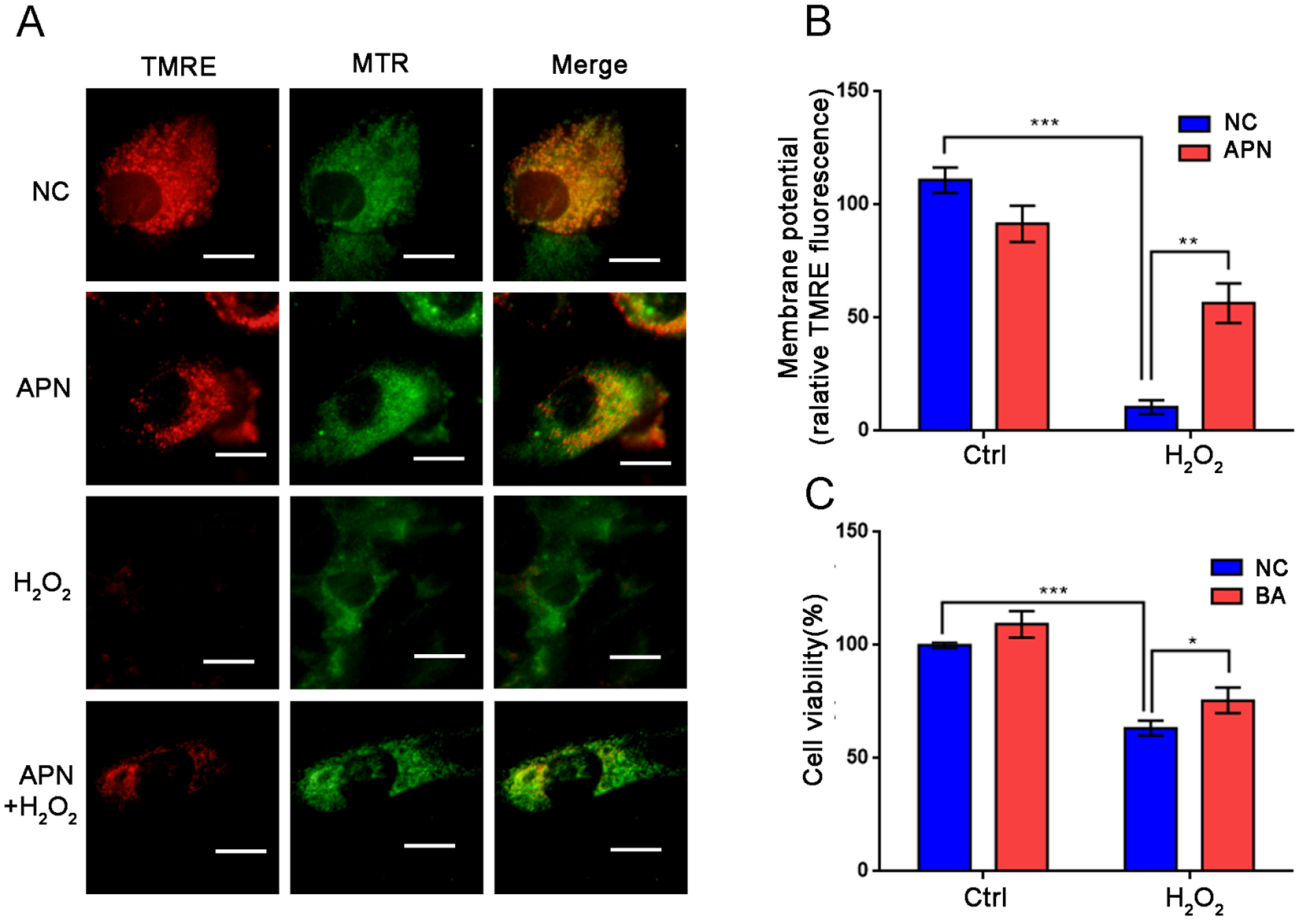
Effect of APN on H_2_O_2_-induced mitochondrial membrane potential. (**A**) C2C12 cells were transfected with 30 μg/mL APN, and control cells were treated with 5 mM H_2_O_2_ for 1 h. Representative fluorescence images of TMRE and MitoTracker Green FM (MTR) are shown. Scale bar, 5 μm. (**B**) Quantification of TMRE signals in cells before and after H_2_O_2_ treatment (mean ± SEM; n = 100 cells from 3 independent experiments, **p < 0.01, ***p < 0.001). (**C**) Influence of BA on H_2_O_2_-induced cell viability. C2C12 cells were pretreated with 15 μM BA for 1 h and then incubated with or without 5 mM H_2_O_2_ for 1 h. Cell viability was evaluated using a Cell Counting Kit-8. The data are expressed as the percentage of mean values ± SEM of experiments performed in triplicate (*p < 0.05, ***p < 0.001).

As previously reported, mitochondria represent both a major source of ROS and the primary target of ROS damage. Excess ROS generated by mitochondria lead to mitochondrial permeability transition pores opening, which causes depolarization of mitochondrial membrane potential^8^. We next investigated the effects of APN on the mitochondrial membrane potential (ΔΨm). The mitochondrial membrane potential was quantified by tetramethylrhodamine ethyl ester (TMRE), a cationic fluorescent stain that accumulates inside the mitochondrial matrix depending on the membrane potential. Mitochondrial morphology was observed by MitoTracker Green FM, a mitochondrial dye that fluoresces independently of the mitochondrial membrane potential. H_2_O_2_ treatment resulted in a significant reduction in the membrane potential in C2C12 cells (Fig. 3A,B). However, APN pretreatment caused a marked increase in the membrane potential in H_2_O_2_-treated cells (Fig. 3A). APN has the same effect on TMRE fluorescence reduction as assessed by FACS analysis (Fig. S1). Bongkrekic acid (BA) is an inhibitor of adenine nucleotide translocase, which is a component of the MPTP complex. Therefore, BA prevents mitochondrial depolarization. Interestingly, similar to the result obtained with APN, BA pretreatment increased cell viability in response to H_2_O_2_ treatment (Fig. 3C). Thus, APN may act as an inhibitor of MPTP to prevent mitochondrial depolarization.

### APN partially blocks the colocalization of mitochondria with autophagosomes/lysosomes

**Figure 4:**
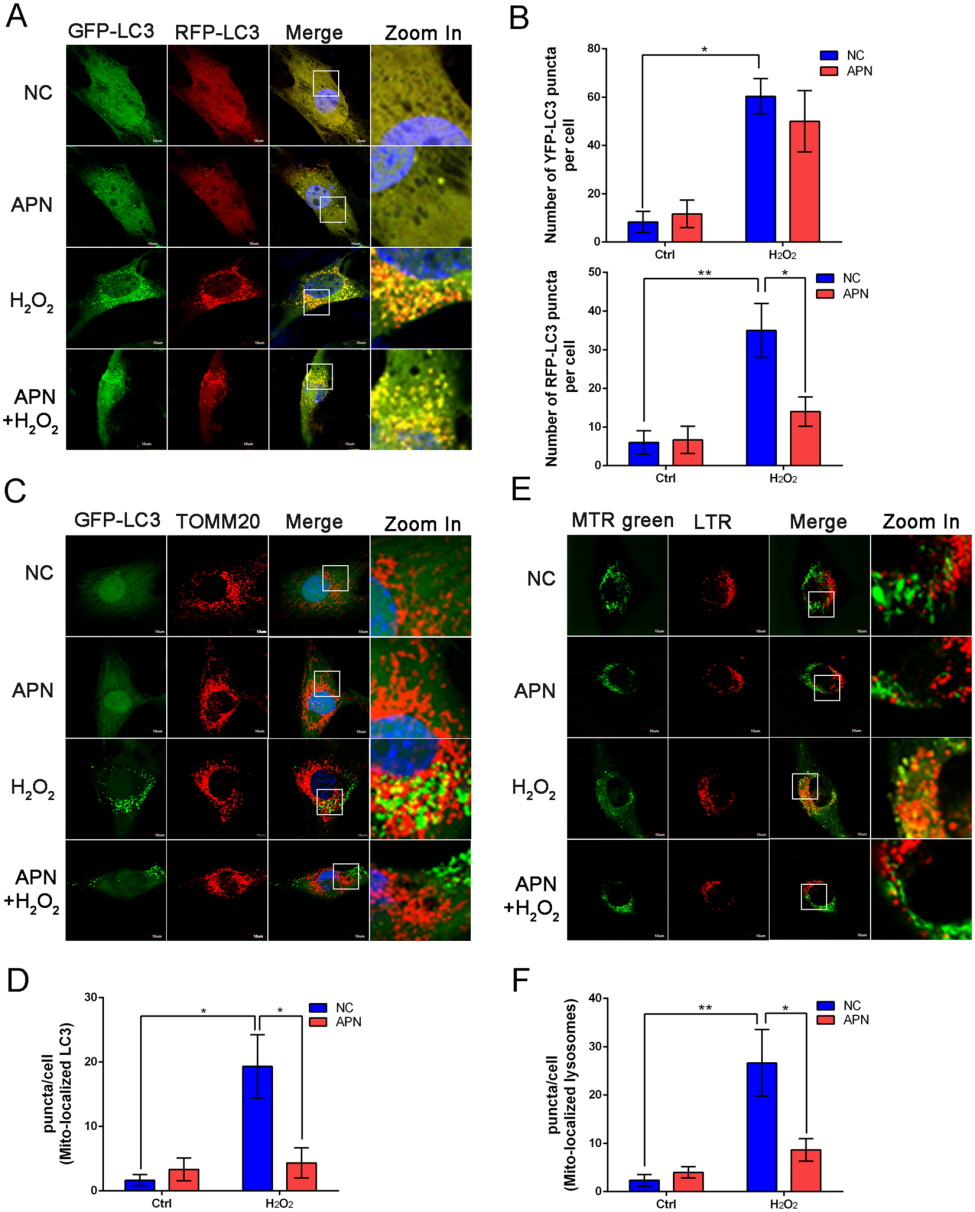
APN partially blocks the colocalization of mitochondria and autophagosomes/lysosomes. (**A**) C2C12 cells were transfected with Ad-mRFP-GFP-LC3 for 24 h, followed by incubation with 30 μg/mL APN for 24 h, and finally incubated with or without 5 mM H_2_O_2_ for 1 h. The distribution of YFP (RFP + GFP + )- or RFP (RFP + GFP−)-LC3 puncta was visualized by confocal microscopy. Scale bar, 10 μm. (**B**) Quantitative analysis of YFP- and GFP-LC3 puncta, which represent autophagosomes and autolysosomes, respectively (mean ± SEM; n = 100 cells from 3 independent experiments, *p < 0.05, **p < 0.01). (**D**) C2C12 cells transfected with GFP-LC3 were treated with or without 5 mM H_2_O_2_ for 1 h, followed by staining with MitoTracker Red for mitochondria and confocal microscopy to visualize GFP-LC3 puncta. Scale bar, 10 μm. (**E**) Quantification of GFP-LC3 punctate structures associated with mitochondria (TOMM20) described in (**D**) (mean ± SEM; n = 100 cells from 3 independent experiments, *p < 0.05). (**E**) Representative fluorescence images of MitoTracker Green FM (MTR green) and LysoTracker Red (LTR) in C2C12 cells treated with or without 5 mM H_2_O_2_ for 1 h. Scale bar, 10 μm. (**F**) Quantitative analysis of cells that contained fragmented mitochondria-localized lysosomes from three independent experiments (mean ± SEM; n = 100 cells, *p < 0.05, **p < 0.01).

To assess dynamic autophagy regulation after mitochondrial depolarization, C2C12 cells were transfected with an adenovirus expressing mRFP-GFP-LC3. In the transfected cells, autophagosomes are labeled green and red (merged in yellow), and autolysosomes are only labeled red because GFP signals are rapidly quenched under conditions of low lysosomal pH. As shown in Fig. 4A,B, we observed a markedly increased number of autophagosomes and autolysosomes after H_2_O_2_ treatment, whereas autolysosomes puncta accumulation was decreased in C2C12 cells upon APN pretreatment. To assess the potential colocalization between mitochondria and autophagosomes, C2C12 cells were transfected with GFP-LC3, followed by staining with MitoTracker Red (TOMM20) for mitochondria. Colocalization between mitochondria and of LC3-labelled autophagosomes was present in C2C12 cells after H_2_O_2_ treatment, whereas APN markedly decreased the colocalization (Fig. 4C,D). Damaged mitochondria are wrapped into autophagosomes and delivered to lysosomes for degradation; thus, we assessed the colocalization of lysosomes and mitochondria after mitochondrial depolarization. Lysosomes and mitochondria were labeled with LysoTracker Red and MitoTracker Green FM, respectively. Similarly, APN decreased the colocalization of mitochondria with lysosomes in response to H_2_O_2_ treatment (Fig. 4E,F).

### APN suppresses H_2_O_2_-induced mitophagy

**Figure 5:**
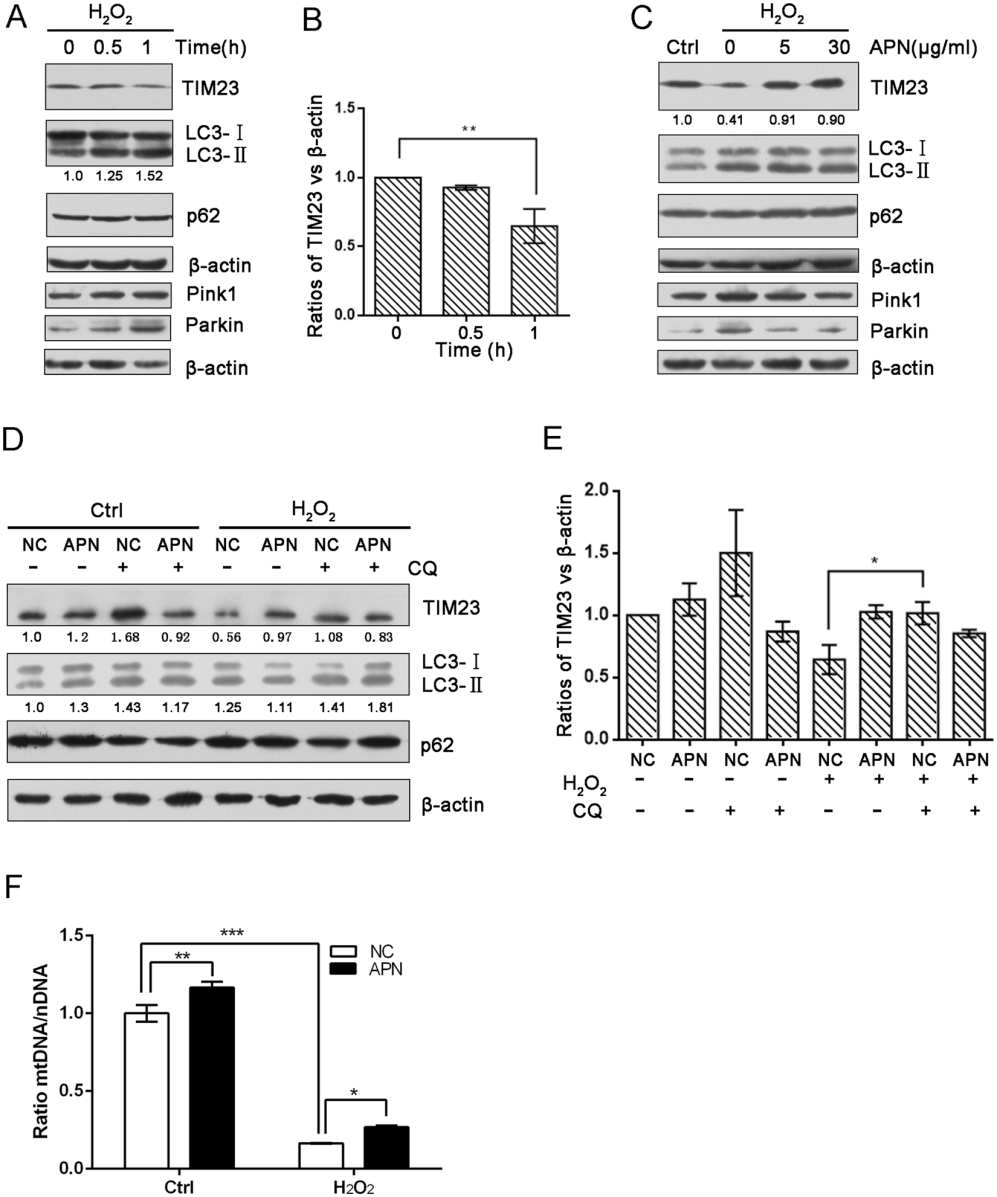
APN suppresses H_2_O_2_-induced mitophagy. (**A**) C2C12 cells were treated with 5 mM H_2_O_2_ for the indicated periods. Samples were collected for western blotting to analyze the expression of TIM23, LC3, p62, Pink1, Parkin and β-actin. (**B**) The densitometric ratios of mitochondrial marker proteins and LC3 were quantified using Image J software. The analysis of the TIM23/β-actin ratios from the immunoblots is shown (data from three independent experiments). (**C**) C2C12 cells pretreated or not with various concentrations of APN followed by treatment with 5 mM H_2_O_2_ for 1 h. Western blotting was performed to analyze the levels of TIM23, LC3, p62, Pink1, Parkin and β-actin. (**D**) C2C12 cells pretreated with APN or not were exposed to CQ (60 μM) for 4 h, followed by incubation with or without 5 mM H_2_O_2_ for 1 h. Western blotting was performed to analyze the levels of TIM23, LC3, p62 and β-actin. (**E**) Image J densitometric analysis of the TIM23/β-actin ratios from the immunoblots is shown (data from three independent experiments) (*p < 0.05, **p < 0.01). (**F**) Quantification of mitochondrial DNA copy number in NC or APN pretreated cells treated with H_2_O_2_ (data from three independent experiments, *p < 0.05, **p < 0.01, ***p < 0.001).

Mitophagy can be observed biochemically by measuring the degradation of mitochondrial proteins from the inner membrane. Here, we used the mitochondrial protein TIM23 as an indicator of mitophagy^40, 41^. We evaluated the time course of TIM23, LC3 and p62 protein levels in response to H_2_O_2_ treatment. According to the TIM23 levels, we found a dramatic decrease in mitophagy after H_2_O_2_ treatment (5 mmol/L) in C2C12 cells. H_2_O_2_ administration did not induce significant changes in p62 expression from 0.5 to 1 h; however, the level of LC3 increased over time (Fig. 5A,B). Immunoblotting revealed that pretreatment with various concentrations APN suppressed H_2_O_2_-induced degradation of TIM23 without affecting the LC3-I to LC3-II transition (Fig. 5C). Numerous studies have analyzed specific regulators of mitophagy including Pink1/Parkin in mammalian systems^42–44^. In response to depolarization of the mitochondria due in part to ROS generation, Pink1 is stabilized on the outer mitochondrial membrane and selectively recruits Parkin to damaged mitochondria^45, 46^. Following its recruitment to the damaged mitochondria, Parkin promotes the degradation of diverse mitochondrial membrane via its E3 ubiquitin ligase activity^44, 47^. We evaluated time course of Pink1 and Parkin protein levels in response to H_2_O_2_ treatment. H_2_O_2_ administration (5 mM) induced significant increases in Pink1 and Parkin expression over time, which were in accordance with the increase observed in LC3 expression (Fig. 5A). Pretreatment with various concentrations APN suppressed increases of Pink1 and Parkin (Fig. 5C). To demonstrate that mitochondria were degraded by an autophagic process, we treated C2C12 cells with the lysosomotropic inhibitor chloroquine diphosphate (CQ) to prevent autophagosome-lysosome fusion. CQ markedly reversed the H_2_O_2_-induced loss of TIM23 in control cells without significantly affecting TIM23 levels in APN-treated cells. These results suggest that APN inhibits H_2_O_2_-induced mitochondrial degradation by autophagy (Fig. 5D,E). To analyze changes in the number of mitochondria as a consequence of mitophagy induced by H_2_O_2_ treatment, we examined alterations in the mitochondrial DNA copy number by real-time PCR. As observed in Fig. 5F, H_2_O_2_-induced loss of mtDNA was significantly prevented by APN.

### APN inhibits H_2_O_2_-induced apoptosis

**Figure 6:**
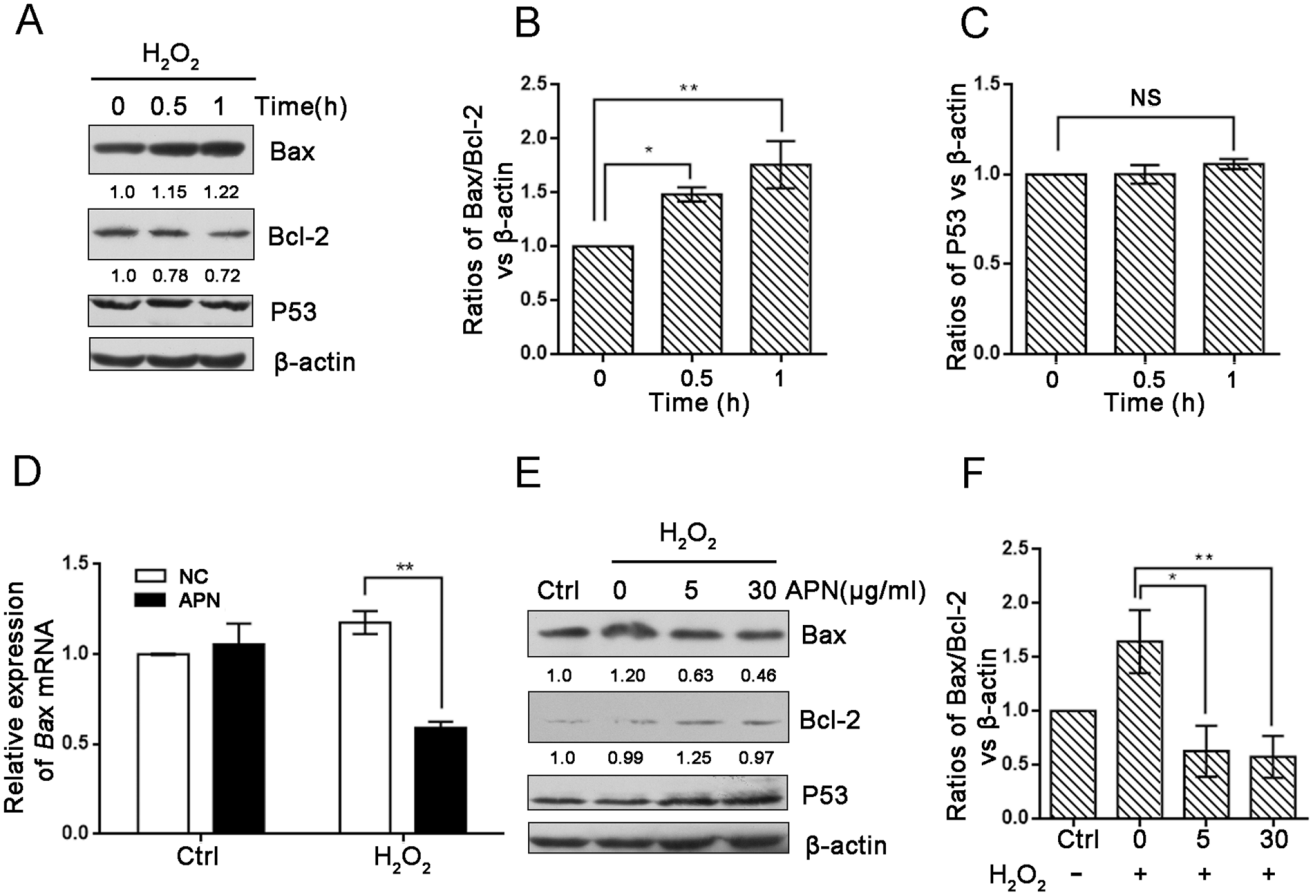
APN inhibits H_2_O_2_-induced apoptosis. (**A**) C2C12 cells were treated with 5 mM H_2_O_2_ for the indicated periods. Samples were collected for western blotting to analyze the expression levels of Bax, Bcl–2, P53 and β-actin. (**B**,**C**) The densitometric ratios of Bax, Bcl-2 and P53 were quantified using Image J software. The analysis of the (Bax/Bcl-2)/β-actin and P53/β-actin ratios from the immunoblots is shown (data from three independent experiments). (**D**) The relative expression of Bax in C2C12 cells pretreated or not with 30 μg/mL APN followed by treatment with 5 mM H_2_O_2_ for 1 h. (**E**) C2C12 cells pretreated or not with various concentrations of APN followed by treatment with 5 mM H_2_O_2_ for 1 h. Western blotting was performed to analyze the levels of Bax, Bcl-2, P53 and β-actin. (**F**) Image J densitometric analysis of the (Bax/Bcl-2)/β-actin ratios from the immunoblots is shown (data from three independent experiments) (*p < 0.05, **p < 0.01).

Accumulating evidence has demonstrated that APN prevents oxidative stress-induced apoptosis. Therefore, we sought to determine the molecular mechanism of APN in cell death induced by H_2_O_2_. Higher Bax and lower Bcl-2 levels were detected in H_2_O_2_-induced cells compared with controls (Fig. 6A,B); however, no significant changes in P53 expression were found (Fig. 6C). We performed quantitative RT-PCR to examine the mRNA levels of Bax. Interestingly, APN pretreatment significantly reduced the transcript levels of Bax in H_2_O_2_-induced cells compared with controls (Fig. 6D). Immunoblotting analysis revealed that various concentrations of APN resulted in potent downregulation of Bax/Bcl-2 protein expression, which also determined the apoptotic potential in C2C12 cells (Fig. 6E,F).

## Discussion

ROS can oxidize cellular components and thus pose a threat to cell integrity. Several defense mechanisms have been demonstrated to protect cells against oxidative stress; such mechanisms include removal of specific proteins by the ubiquitin–proteasome system, upregulation of antioxidants and elimination of damaged organelles by autophagy^4^. These defense systems are compromised in conditions of excess ROS, which ultimately leads to cell death through various overlapping signaling pathways and cascades^3^. In this study, we investigated whether APN exerted protective effects against oxidative stress-induced cytotoxicity. Our results indicated that APN significantly attenuated H_2_O_2_-induced cytotoxicity in C2C12 cells, supporting that APN exerted antioxidant properties. Moreover, APN upregulated Nrf2 and HO-1 protein expression in a concentration-dependent manner. Therefore, we presumed that APN might activate the Nrf2/HO-1 pathway to eliminate the enhanced ROS generation induced by H_2_O_2_, thereby reducing H_2_O_2_-induced apoptosis.

Autophagy has an essential role in differentiation and development and has been associated with myopathies, neurodegenerative diseases, pathogen infections and cancer^4^. When survival programs fail, death mechanisms are activated in response to oxidative stress. In addition to its role in cell survival, autophagy has recently been reported to induce cell death^48^, which is referred to as type II cell death^49–51^. In Parkinson’s disease, for example, oxidation of dopamine leads to oxidative stress, autophagy and cell death. Therefore, autophagy has a dual role in the response to oxidative stress. In our study, excessive ROS induced autophagy and cell death. Moreover, depolarized mitochondria, which are unable to reestablish membrane potential, are excluded and targeted for mitophagy. As recently reported, it is possible that both ROS and depolarization are necessary for the induction of mitophagy^22^.

In this study, we used mouse-derived C2C12 myoblasts as an *in vitro* model to determine the role of APN in mitophagy and apoptosis. Adiponectin normally circulates abundantly in the concentration range of 5 to 30 μg/mL. The dose of APN used can be considered in a physiological level. Interestingly, we showed that APN pretreatment suppressed H_2_O_2_-induced mitophagy by inhibiting the degradation of mitochondrial TIM23 protein in addition to decreasing global autophagy. Although p62, which exhibited no significant changes in our study, is widely used as a biochemical marker for general autophagy, it is not a reliable marker for H_2_O_2_-induced autophagy in C2C12 cells. We found that Pink1 expression increased concomitant with Parkin induction under H_2_O_2_ treatment. Expression of lipidated form of LC3 was also elevated, suggesting that Parkin mediated mitochondrial degradation is performed, in part, by mitophagy, although proteasome could be also implicated due to the E3 ubiquitin ligase activity of Parkin. Besides, APN administration to H_2_O_2_-treated cells suppressed increases of Pink1 and Parkin. These findings suggest that APN inhibits Parkin E3 ubiquitin ligase and partially blocks the colocalization of mitochondria and autophagosomes/lysosomes. We further demonstrated that APN inhibited C2C12 cell apoptosis by reducing the transcript and protein levels of Bax. However, these results differ from those reported for high-fat diet-induced oxidative stress mouse models, in which APN administration stimulates autophagy and reduces oxidative stress to enhance insulin sensitivity^52^. Similar to our results, APN ameliorates H_2_O_2_-induced autophagy in cardiomyocytes, moreover, loss of APN enhances autophagy in response to Ang-II infusion *in vivo* ^30^. Therefore, APN modulation of mitophagy seems to be context and cell-type dependent, which is similar to its effects on other signaling pathways^53^.

**Figure 7:**
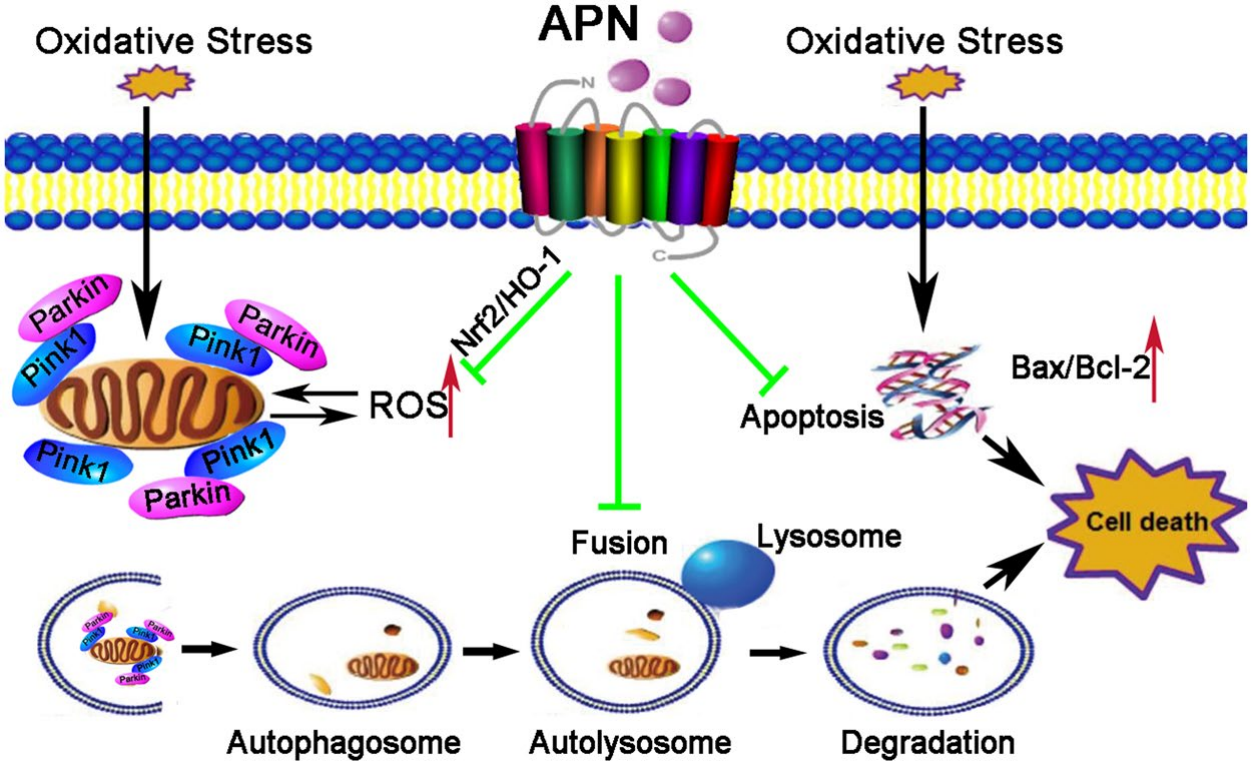
Proposed model of action of APN on oxidative stress. H_2_O_2_ administration to C2C12 cells causes reductions in mitochondrial membrane potential, ROS production and apoptosis. High levels of ROS and depolarized mitochondria induced mitophagy. APN pretreatment suppressed the over-production of ROS by activating the Nrf2/HO-1 pathway, protecting mitochondria, inhibiting Parkin E3 ubiquitin ligase and modulating mitophagy by partially blocking the colocalization of mitochondria with autophagosomes/lysosomes. In addition, APN downregulates the transcript levels of both Bax and Bax/Bcl-2 protein induced by oxidative stress.

In summary, the results of the present study indicated that APN inhibited oxidative stress-induced cellular damage in C2C12 cells. This beneficial effect was closely associated with its potential to eliminate excess ROS accumulation, to prevent mitochondrial depolarization, to modulate autophagy and to suppress apoptosis (Fig. 7). In other experiment, APN protects against acetaminophen-induced oxidative stress, mitochondrial dysfunction and acute liver injury by promoting autophagy in mice^54^. Although further studies are necessary to better understand the molecular mechanisms that are involved, our experiments confirm the antioxidant potential of APN and underscore its protective role in skeletal muscle diseases related to oxidative stress.

The reciprocal interactions between autophagy and oxidative stress should also be further investigated, for example, using APN-deficient animal models or autophagy-deficient animal models. Moreover, further studies in rodent models, large humanoid animals and clinical investigations are warranted to validate these findings *in vitro* models. However, other cellular events, such as ROS, precede changes in mitophagy and autophagy itself may still be viewed as a doubled-edged sword and the temporal nature of the process can lead to distinct cellular consequences.

## Methods

### Cell culture and reagents

C2C12 myoblasts obtained from the Cell Resource Center (Peking Union Medical College) were grown in Dulbecco’s modified Eagle’s medium (DMEM) supplemented with 10% heat-inactivated fetal bovine serum (HyClone, GE) and 100 μg/mL penicillin/streptomycin antibiotics in an incubator with 5% CO_2_ at 37 °C. Recombinant mouse full-length APN was synthesized by Gencreate (Wuhan, China) and dissolved in phosphate-buffered saline (PBS) at a concentration of 500 μg/mL. Hydrogen peroxide (H_2_O_2_) was purchased from Solarbio (Beijing, China). Bongkrekic acid (BA) was purchased from Sigma. Chloroquine diphosphate (CQ) was purchased from InvivoGen.

### Cell viability assay

Cells were seeded in 96-well culture plates (4 × 10^4^ cells per well) and incubated for 24 h. The cells were treated with the indicated concentrations of APN in the presence or absence of H_2_O_2_ for the indicated times. The effect of APN on the inhibition of cell growth was measured as the percentage of cell viability, which was assessed using a Cell Counting Kit-8 (Dojindo, Kumamoto, Japan) as follows. Ten microliters of Cell Counting Kit-8 solution was added to the medium, and cells were incubated for 1 h in a humidified 5% CO_2_ atmosphere; the amount of orange formazan staining was calculated by measuring the absorbance at 450 nm using a microplate reader.

### ROS measurement

C2C12 myoblasts were plated in 12-well plates at a density of 2 × 10^5^ cells per well and treated with or without 30 μg/mL APN for 24 h. After treatment, the cells were incubated with 10 μM 2′,7′-dichlorofluorescein diacetate (DCFH-DA, Beyotime, S0033) for 30 min at 37 °C in the dark and then incubated with or without 5 mM H_2_O_2_ for 30 min. The cells were immediately analyzed using an Accuri C6 (BD, CA, USA). A total of 10,000 cells per sample were acquired and analyzed using FlowJo software (Tree star, Ashland, OR).

### Annexin V–propidium iodide assay

Apoptosis was assessed using an Annexin V-FITC Apoptosis Analysis Kit (Tianjin Sungene Biotech Co., Ltd.). C2C12 myoblasts were seeded in a 12-well plate at a density of 2 × 10^5^ cells per well and treated with or without 30 μg/mL APN for 24 h. Then, the cells were treated in the presence or absence of 5 mM H_2_O_2_ for 30 min. The cell pellets were resuspended in 100 μL buffer with 5 μL annexin V-FITC for 10 min and then incubated with 5 μL propidium iodide for 5 min at room temperature in the dark. A total of 500 μL buffer was added, and the cells were immediately analyzed using an Accuri C6 (BD, CA, USA). A total of 10,000 cells per sample were acquired, and percentage of cell death was analyzed.

### Measurement of mitochondrial membrane potential

Mitochondrial membrane potential was measured using the fluorescent strain TMRE (Sigma, 87917). C2C12 myoblasts treated with or without APN were treated with or without 5 mM H_2_O_2_ for 30 min and then incubated with TMRE (50 nM) and MitoTracker Green FM (200 nM) (Beyotime, C1048) for 30 min at 37 °C. The relative fluorescence intensity of TMRE was quantified using Image-Pro Plus 6 Windows Software (Media Cybernetics, USA). Or C2C12 myoblasts treated with or without APN were treated with or without 5 mM H_2_O_2_ for 30 min and then incubated with TMRE (50 nM) for 30 min at 37 °C. A total of 200 μL PBS/0.2% BSA was added, and the cells were immediately analyzed using an Accuri C6 (BD, CA, USA). A total of 10,000 cells per sample were acquired and analyzed using FlowJo software (Tree star, Ashland, OR). The data shown are representative of three independent experiments.

### Analysis of autophagic flux

C2C12 myoblasts stably transfected with Ad-mRFP-GFP-LC3 (Hanbio, Shanghai, China) were seeded on glass cover slips. Twenty-four hours after transfection, the cells were treated with or without APN (30 mg/mL up to 24 h). The cells were then stimulated with H_2_O_2_ for 30 min to induce autophagy. Coverslips were washed twice with PBS 1X and mounted on glass slides with fluorescent mounting medium Fluoroshield™ with DAPI (eBioscience) and visualized in an Olympus FV1000 confocal microscope.

### Immunofluorescence and laser confocal imaging

C2C12 myoblasts stably transfected with Ad-GFP-LC3 (Hanbio, Shanghai, China) were seeded on glass cover slips. Twenty-four hours after transfection, the cells were treated with or without APN (30 μg/mL up to 24 h). The cells were then stimulated with H_2_O_2_ for 30 min. After that, cells were fixed for 15 minutes with 4% paraformaldehyde and washed twice with PBS 1X. Cells were blocked and permeabilized with PBS 1X + 0.2% Triton X-100 for 15 minutes at room temperature. After washing twice with PBS 1X, cells were incubated with a rabbit monoclonal TOMM20 antibody (Abcam) diluted 1:250 in 5% BSA O/N at 4 °C and washed twice with PBS 1X followed by incubation with a secondary anti-rabbit IgG antibody, conjugated to Alexa 555 (1:200) for 1 hour at room temperature. Coverslips were washed twice with PBS 1X and mounted on glass slides with fluorescent mounting medium Fluoroshield™ with DAPI (eBioscience) and visualized in an Olympus FV1000 confocal microscope.

To determine colocalization between mitochondria and lysosomes, C2C12 myoblasts were loaded with 200 nM MitoTracker Green FM (Beyotime, C1048) and 50 nM LysoTracker Red (Beyotime, C1046) for 30 min at 37 °C. Cell images were obtained using an Olympus FV1000 confocal microscope. The colocalization of mitochondria and lysosomes was analyzed according to the number of mitochondria-localized lysosomes.

### qRT-PCR gene expression analysis

Total RNA was isolated with TRIzol reagent (Invitrogen, Carlsbad, USA) according to the manufacturer’s instructions. Relative gene expression was detected using quantitative RT-PCR. Total RNA was reverse-transcribed using a reverse transcription kit (Takara, Japan). Quantitative PCRs were performed using SYBR Green PCR Master Mix (Takara, Japan) according to the manufacturer’s instructions. Relative mRNA levels were normalized to Gapdh expression. The primer sequences of the mouse gene are as follows:

Bax forward primer, TGAAGACAGGGGCCTTTTTG;

Bax reverse primer, AATTCGCCGGAGACACTCG (GenBank # NC_000073.6).

Gapdh forward primer, CCATGTTCGTCATGGGTGTGAACCA;

Gapdh reverse primer, GCCAGTAGAGGCAGGGATGATGTTC (GenBank # NC_000072.6).

### Western blot analysis

Western blot analysis was conducted to detect the expression of TIM23, LC3, p62, Bax, Bcl-2, P53, Nrf2, HO-1, Pink1, Parkin and β-actin protein in C2C12 myoblasts. LC3 rabbit monoclonal antibody and p62 rabbit polyclonal antibody (1:1000 dilution) were purchased from Cell Signaling (Beverly, MA, USA). Bax, Bcl-2, Nrf2, HO-1 and Parkin rabbit monoclonal antibodies (1:1000) were purchased from Abcam. Pink1 rabbit polyclonal antibody (1:1000) was purchased from ABclonal Technology. TIM23 and P53 rabbit polyclonal antibodies (1:1000) were purchased from Proteintech. β-actin mouse monoclonal antibody (1:2000) was purchased from Sungene. RIPA lysis buffer with 1% phosphatase inhibitor cocktail and 1 mM PMSF was used for the whole-cell lysates. Protein concentrations were detected via the BCA method (Biomed, Beijing, China) according to the manufacturer’s instructions. Equal amounts of protein were separated via 12% SDS-PAGE, transferred to PVDF membranes, and subsequently detected using various primary antibodies. The membranes were incubated for 1.5 h at room temperature with the appropriate secondary HRP-conjugated antibody (1:5000; Tianjin Sungene Biotech) and visualized using an ECL detection kit (Millipore Corporation, Billerica, MA, USA). The densities of specific bands were quantified using ImageJ software.

### Mitochondrial DNA content

Genomic DNA was isolated with TIANamp Genomic DNA Kit (TIANGEN BIOTECH (BEIJING) CO., LTD) according to the manufacturer’s instructions. Quantitative PCRs were performed using SYBR Green PCR Master Mix (Takara, Japan) according to the manufacturer’s instructions^40^. To quantify the amount of mtDNA present per nuclear genome, we used the following primers^55^:

mtDNA forward primer, CCTATCACCCTTGCCATCAT;

mtDNA reverse primer, GAGGCTGTTGCTTGTGTGAC (GenBank # NC_000085.6).

To quantify nuclear DNA, we used a primer set that detects the Pecam gene:

nuclear DNA forward primer, ATGGAAAGCCTGCCATCATG;

nuclear DNA reverse primer, TCCTTGTTGTTCAGCATCAC (GenBank # NC_000077.6).

### Statistical analysis

The data are presented as the mean values ± SEM of at least three independently repeated experiments. One-way ANOVA followed by Tukey’s HSD post hoc test was used to measure differences between mean values of the different treated groups. *p* < 0.05 was considered significant. The values were analyzed using GraphPad Prism, version 6.0 (GraphPad Software, San Diego, CA, USA).

## Electronic supplementary material

Supplementary figure and legend
