# Supplementary material for: Adiponectin modulates oxidative stress-induced mitophagy and protects C2C12 myoblasts against apoptosis
Source: Sci Rep. 2017 Jun 9;7:3209. doi: 10.1038/s41598-017-03319-2 (PMC5466641; doi:10.1038/s41598-017-03319-2)
Supplement: Supplementary file 1 — Supplementary figure and legend [file 41598_2017_3319_MOESM1_ESM.pdf]

# Adiponectin modulates oxidative stress-induced mitophagy and protects C2C12 myoblasts against apoptosis

Yinghui Ren [a, †], Yan Li [a, †], Jun Yan [b, †], Mingkun Ma [a,c], Dongmei Zhou [a], Zhenyi Xue [a], Zimu Zhang [a], Hongkun Liu [a], Huipeng Yang [a], Long Jia [a], Lijuan Zhang [a], Qi Zhang [a], Shuqin Mu [b, *], Rongxin Zhang [a,d, *] and Yurong Da [a, *]

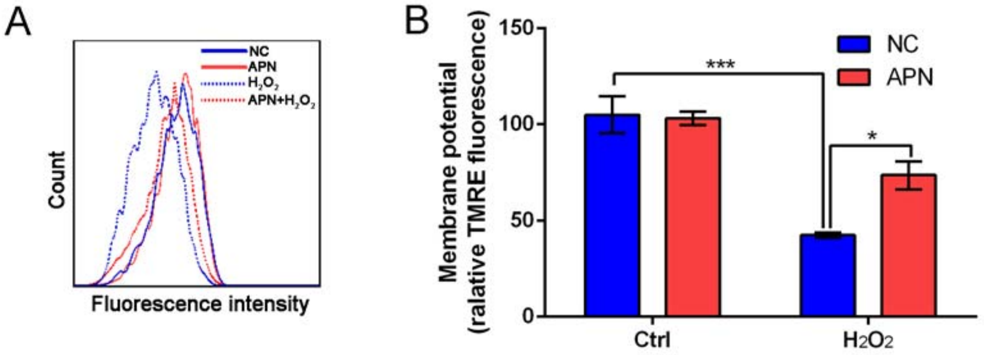


**Supplementary figure 1. Effect of APN on $H_2O_2$-induced mitochondrial membrane potential.** (**A**) C2C12 cells were transfected with 30 μg/mL APN, and control cells were treated with 5 mM $H_2O_2$ for 30 min and then incubated with TMRE (50 nM) for 30 min at 37°C. TMRE fluorescence intensity was measured using a flow cytometer. (**B**) The results are presented as the mean ± SEM values obtained from three independent experiments (*$p$<0.05, ***$p$<0.001).
